# Developing a Dyadic Immersive Virtual Environment Technology Intervention for Persons Living With Dementia and Their Caregivers: Multiphasic User-Centered Design Study

**DOI:** 10.2196/66212

**Published:** 2025-05-21

**Authors:** Elizabeth A Rochon, Ayush Thacker, Mirelle Phillips, Christine Ritchie, Ana-Maria Vranceanu, Evan Plys

**Affiliations:** 1 Center for Health Outcomes and Interdisciplinary Research Department of Psychiatry Massachusetts General Hospital Boston, MA United States; 2 Mongan Institute Center for Aging and Serious Illness and the Division of Palliative Care and Geriatric Medicine Department of Medicine Massachusetts General Hospital Boston, MA United States; 3 Studio Elsewhere Brooklyn, NY United States; 4 Harvard Medical School Boston, MA United States

**Keywords:** dementia, caregivers, dyads, technology, design, immersive

## Abstract

**Background:**

Persons living with dementia and their caregivers experience frequent emotional health challenges. Across the illness spectrum, engaging in shared pleasant activities is an important feature of well-being for persons living with dementia–caregiver dyads. Under the umbrella of virtual reality, immersive virtual environment technology (IVET) offers artificial sensory experiences and shows promise in this population. IVET development benefits from a user-centered design approach, and as an emerging field, preliminary testing of safety, usability, and engagement for person living with dementia–caregiver dyads is required.

**Objective:**

We aimed to develop a preliminary IVET intervention for psychosocial health among person living with dementia–caregiver dyads. In doing so, we highlight design considerations and user preferences to ensure the safety and usability of technology-based interventions in the context of dementia.

**Methods:**

We engaged 10 clinicians, 8 caregivers, and 3 persons living with dementia in 5 rounds of focus groups to evaluate the safety and usability of preliminary intervention features. Following prototype development, we engaged caregivers and persons living with dementia (n=9 dyads) in beta testing workshops to observe real-time user interaction with the intervention and guide refinements. Rapid data analysis was used to extract themes relevant to intervention development.

**Results:**

The following themes emerged from focus groups to inform prototype development: (1) designing flexibly to allow users to tailor the intervention experience to their own environmental context and circumstance, (2) designing with the dyad’s clinical and relational needs in mind, and (3) accounting for illness and aging-related challenges in design. The following themes emerged from workshops to inform prototype refinements: (1) increasing user support through more feedback and (2) increasing variety of visual and auditory feedback.

**Conclusions:**

Using user feedback throughout the development process, we developed a prototype of an IVET intervention, Toolkit for Experiential Well-Being in Dementia (the Isle of TEND), tailored to the needs of persons living with dementia and their caregivers. Our prototype uses specific design features to promote safety, usability, and engagement in the context of dementia. Future feasibility testing of the intervention is warranted.

**International Registered Report Identifier (IRRID):**

RR2-10.2196/52799

## Introduction

### Background

Throughout the course of dementia, emotional health challenges (eg, depression, negative affect, and worry) are common among both persons living with a dementia diagnosis and their informal caregivers (eg, family members and friends) [1,2]. Persons living with dementia and their caregivers present with different emotional needs throughout the course of dementia [3,4]; however, maintaining relationship quality within the context of changing interpersonal dynamics remains an important component of emotional health for both members of the person living with dementia–caregiver dyad [5].

Dyadic research is gaining popularity within the fields of emotional health and dementia care [6,7]. Dyadic research highlights the impact of interpersonal relationships and the transmission of emotional states between members of the person living with dementia–caregiver dyad, which influence the emotional health of each member [8]. For example, perceived relationship strain by the persons living with dementia is associated with increased emotional distress in their caregivers and vice versa [9,10]. Alternatively, positive affective states can be shared among dyad members (eg, laughter), increasing emotional health for both dyad members [11]. Indeed, relationships and the emotional health needs of persons living with dementia and caregivers often change throughout the course of dementia, especially in advanced stages, as cognitive and communication limitations are more common [4,12]. However, despite changes, the need for positive experiences shared between persons living with dementia and caregivers remains common and relates to fewer behavioral and psychiatric symptoms among persons living with dementia and less stress among caregivers [13]. Thus, shared pleasant activities are an important component of emotional health among person living with dementia–caregiver dyads that can be targeted at any stage of illness.

As dementia advances and cognitive impairments become more pronounced, identifying shared activities that can promote emotional health for both persons living with dementia and caregivers may become more challenging [14-16]. Indeed, many dyadic interventions in the context of dementia are concerned with systematic approaches to identifying and engaging in shared activities [17]. For example, the tailored activity program intervention helps dyads identify and engage in personalized shared activities to reduce agitation among persons living with dementia and improve well-being among caregivers [18,19]. However, many of these shared activity interventions rely heavily on caregivers to plan and initiate activities, which may reduce shared positive affective states due to the caregiver-care recipient role, rather than equitable activity engagement [11]. For example, caregivers may find themselves in the role of an “instructor” or “supervisor,” rather than fully engaging in and receiving the emotional benefits of an activity. Therefore, designing interventions that allow both persons living with dementia and caregivers to fully engage in shared activities may create a positive emotional experience for each dyad member, contributing to their emotional health. Technology may aid this endeavor by creating interactive experiences that minimize the need for caregiver initiation (ie, using a technology platform that engages and guides both members of the dyad through an activity, rather than relying on the caregiver to lead the activity). Immersive virtual environment technology (IVET) is one such option for engaging person living with dementia–caregiver dyads in an intervention together.

IVET exists under the umbrella of virtual and augmented reality [20] and is defined as an artificial sensory experience that users actively engage with as if it were a real experience [21]. IVET promotes active engagement between users and computer-simulated environments through multisensory user-driven experiences [13,21,22]. These methods align well with existing approaches to promoting positive affect among persons living with dementia (ie, multisensory stimulation and personalized activities) [23]. IVETs often follow the 3 I’s design framework [24], which suggests that user engagement is achieved through (1) immersion (ie, creating a feeling of presence within the virtual environment), (2) interaction (ie, creating a sense of agency and control over the virtual environment), and (3) imagination (ie, the potential to create new experiences within the virtual environment). In nondementia populations, IVET interventions have demonstrated positive effects on emotional health outcomes, such as positive affect and emotional health (eg, depression and stress) [25]. Importantly, IVET interventions vary in terms of the level of immersion, categorized as low (ie, screen-based intervention), medium (ie, cave automatic virtual environments and surrounding projection screens), or high (ie, head-mounted displays for isolation from physical reality) [25]. In nondementia populations, medium immersion produces the greatest results on emotional health, with little difference observed between low and high immersion [25]. However, given the potential safety risks, such as cybersickness, falls, and agitation associated with higher levels of immersion in the context of dementia [21,26], IVET with low immersion may be suitable for this population. Indeed, most IVET interventions tested within the context of dementia use lower levels of immersion [27].

IVET interventions allow for multiple users to engage jointly (eg, persons living with dementia and their caregivers participate in shared activities) [21]. However, existing interventions have been tested primarily among persons living with dementia and caregivers individually [27-30]. For persons living with dementia, IVET interventions have demonstrated feasibility and usability, primarily targeting cognitive rehabilitation, exercise, or spatial navigation training [27]. For caregivers, IVET interventions have demonstrated feasibility and usability, often focused on caregiver education and skills [31,32]. Fewer IVET interventions have been developed for person living with dementia–caregiver dyads to participate in jointly (ie, dyadically), with a focus on shared positive experiences for emotional health. SENSE-GARDEN is an example of a dyadic IVET intervention, which creates an immersive environment based on preferred autobiographical experiences, such as music, images, videos, and personal media (eg, family photos), for persons living with dementia who have moderate to severe cognitive limitations, allowing them to navigate along with their caregivers. The intervention is designed to support reminiscence between the persons living with dementia and caregivers in the present moment. Notably, caregivers have an active role in personalizing the information in SENSE-GARDEN but receive a lower “active dose” of the intervention (ie, fewer caregiver-focused experiences). Rather, caregiver benefit is conceptualized as arising from positive interactions with persons living with dementia. A clinical trial is currently underway for this intervention [33].

A major challenge to designing dyadic IVET interventions in the context of dementia is addressing cognitively mismatched dyads, which is more common as dementia advances. Promoting shared engagement among person living with dementia–caregiver dyads across the illness spectrum must embrace the 3 I’s of design (ie, immersion, interaction, and imagination) for dyads with potentially different cognitive ability levels. While challenging from a design perspective, if achieved, this type of intervention would be an invaluable opportunity for dyad members to truly engage in shared activities together (ie, not initiated by the caregiver), helping them to briefly abandon the caregiver-care recipient dynamic and, in doing so, derive relational and emotional benefits. These types of more equitable interactions may promote positive affect and interpersonal connection among both persons living with dementia and caregivers, dignity for the persons living with dementia, and positive aspects of caregiving for the caregivers [34]. However, currently, guidelines for designing dyadic IVET interventions to promote shared engagement among person living with dementia–caregiver dyads are lacking, especially for dyads with mismatched cognitive abilities [6,35].

**Figure 1:**
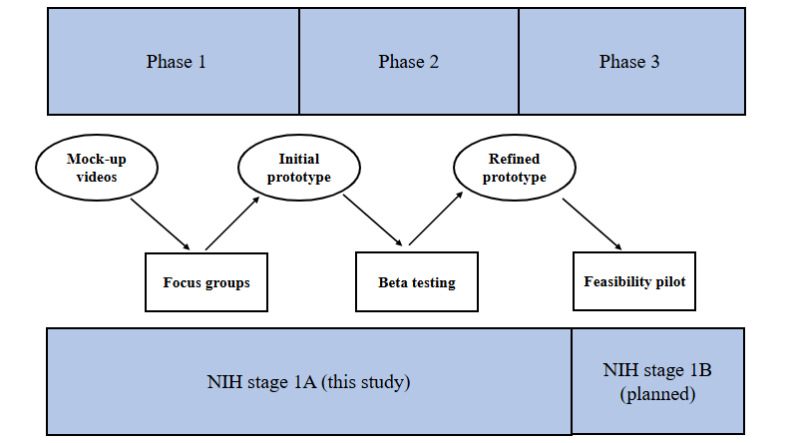
User-centered iterative design to develop immersive virtual environment technology. Circles denote activities initiated by the design team, and rectangles denote activities designed to elicit user feedback. NIH: National Institutes of Health.

Additional work is needed to guide the development of dyadic IVET interventions throughout the course of dementia, especially in more advanced stages. For this endeavor, user-centered design approaches may be particularly important to ensure that interventions have the potential to be acceptable and feasible for both dyad members at various stages of illness. User-centered design is an iterative approach that centers the user experience early and often throughout the intervention development process [36]. The aim of user-centered design is to maximize the feasibility and acceptability of an intervention [37]. This study followed a multiphasic conceptual model of user-centered design rooted in human factors research, as outlined by Witteman et al [38]; refer to Figure 1. In phase 1, design teams engaged users and other knowledge holders with the goal of understanding user needs, goals, and strengths. In phase 2, design teams iteratively developed and refined an initial intervention prototype with user feedback. In phase 3, design teams observed users’ naturalistic interactions with the initial prototype to inform additional refinements [38]. On the basis of user-centered design principles in the context of dementia [39], we prioritized the following factors across each design phase: understandability (ie, the user understands the intervention functions), learnability (ie, the user is able to easily learn the intervention), usability (ie, the user is able to participate in a task with minimal assistance), and safety (ie, tasks do not inadvertently lead to negative affect or other adverse outcomes).

### Purpose

This study aimed to develop a dyadic IVET intervention to support the emotional health of person living with dementia–caregiver dyads. Our design team represents an academic-industry partnership. In this study, we highlight our multiphasic user-centered design process [38], focused on the early stages of intervention development of a dyadic IVET intervention (phases 1 and 2 of user-centered design). First, we present data from focus groups with clinicians, caregivers, and person living with dementia–caregiver dyads to understand user needs, goals, strengths, and usability and safety concerns (phase 1). Then, we discuss the process of iteratively developing and refining an initial intervention prototype based on user feedback from multiple user workshops with person living with dementia–caregiver dyads (phase 2). The resulting dyadic IVET intervention prototype will be ready for pilot testing in a pilot feasibility trial (phase 3). In this study, by describing phases 1 and 2 of user-centered design, we highlight early design considerations for future teams focused on developing IVET or other novel technologies with person living with dementia–caregiver dyads across stages of dementia.

## Methods

### Study Design

This study followed a multiphasic user-centered design framework for the early stages of intervention development, as outlined by Witteman et al [38] and with the following dementia-specific considerations incorporated [39]: (1) understanding user needs, goals, strengths, limitations, and safety concerns; (2) iteratively developing and refining an initial prototype with user feedback; and (3) observing users’ naturalistic interactions with the initial prototype. In phase 1, we conducted 5 focus groups with clinicians, caregivers, and person living with dementia–caregiver dyads to identify preferred activity features as well as design considerations for usability (ie, preferred intervention activities and accommodations) and safety in the context of dementia. The study team determined readiness to move to phase 2 after we believed that we understood major safety risks and could generate enough content for mock-ups of an intervention prototype. In phase 2, we conducted 8 beta testing workshops with person living with dementia–caregiver dyads to inform the development and refinement of an initial prototype. We determined readiness to move to phase 3 when usability concerns were sufficiently addressed, as evidenced by minimal usability issues reported by workshop participants. At the conclusion of phase 2, we had a finalized intervention prototype ready for feasibility testing in phase 3, which is not reported in this study. Phases 1 and 2 map onto stage 1A of the National Institutes of Health (NIH) Stage Model of Behavioral Intervention Development (ie, intervention generation, refinement, modification, and adaptation), and phase 3 maps onto stage 1B (ie, pilot testing) [40,41].

Phases 1 and 2 of intervention development were informed by our intervention conceptual model, as suggested by Rochon et al [42]. Specifically, we aimed to develop an IVET intervention that could promote sustained attention, positive emotions, and active engagement (ie, mechanistic targets) among both persons living with dementia and caregivers, leading to increased relationship satisfaction, decreased distress, and reduced agitation among persons living with dementia, as well as decreased burden among caregivers (ie, outcomes).

### Ethical Considerations

Procedures were approved by the Massachusetts General Hospital Institutional Review Board (2022P001401). The information reported in this manuscript follows the Guidance for the Reporting of Intervention Development standards [43]. Potential clinician participants provided verbal consent before participating in focus group interviews. Caregivers provided verbal consent or e-consent during screening phone calls. A research assistant administered the University of California, San Diego Brief Assessment of Capacity to Consent Questionnaire (UBACC [44]) to persons living with dementia to determine their ability to provide consent. For those unable to provide consent, the persons living with dementia provided assent, and their caregivers provided surrogate consent to participate. All data were deidentified to protect anonymity. In phase 1, clinicians were compensated US $30, and persons living with dementia and caregivers were compensated US $50 each. In phase 2, persons living with dementia and caregivers were compensated US $50 each for their time.

### Participant Recruitment

During phases 1 and 2, this study enrolled three categories of participants: (1) clinicians involved in dementia care, (2) caregivers of persons living with dementia, and (3) person living with dementia–caregiver dyads.

Clinicians were recruited via emails to dementia care clinics within the affiliated health care system. Clinicians were eligible if they (1) self-identified as dementia care providers and (2) were able to participate via Zoom (Zoom Communications). Of the 11 clinicians screened for focus groups, 1 (9%) did not participate due to a scheduling conflict.

Caregivers and dyads were both recruited via research registries (eg, study fliers). Recruitment materials were designed to target caregivers. Interested caregivers responded to the research team to obtain additional information about the study and review eligibility. Caregivers were eligible if they (1) aged >18 years, (2) self-identified as informal caregivers for a person living with dementia (eg, a family member or friend providing support), (3) could participate in English, and (4) had access to a video-enabled device for Zoom participation. Of the 31 caregivers screened for focus groups, 5 (16%) were ineligible due to not identifying as a caregiver of a person living with dementia, 13 (42%) had scheduling conflicts, and 5 (16%) could not be contacted.

For person living with dementia–caregiver dyads, caregivers were targeted using similar recruitment strategies discussed above. Interested caregivers identified and contacted their care recipient with dementia to participate in the study. Then, caregivers and persons living with dementia participated in a screening call together. Using the same eligibility criteria stated in the Participant Recruitment section, caregivers provided verbal consent or e-consent during screening calls. Eligibility criteria for persons living with dementia included (1) having an informal caregiver participating in the study, (2) a self- or caregiver-reported dementia diagnosis, and (3) the ability to participate in an interview conducted in English. The UBACC uses 2 open-ended questions to assess the understanding of informed consent information (eg, “Can you briefly tell me what the purpose of the study is based on what I just read?”); scores range from 0 to 2, with scores <2 indicating lack of capacity to provide consent. The UBACC scores show a strong correlation with global cognition [45], suggesting that lower scores (ie, the inability to provide consent) are indicative of more advanced stages of dementia. Across stages, of the 12 persons living with dementia screened for this study, 8 (67%) scored a 0, a total of 2 (17%) scored 1, and 2 (17%) scored 2 (ie, able to consent independently). Of the 26 caregivers screened for workshops, 1 (4%) was ineligible due to not identifying as a caregiver, 4 (15%) had scheduling conflicts, 3 (12%) declined due to the care partner’s worsening medical status, and 9 (35%) could not be contacted.

### Phase 1: Focus Groups

#### Participants

There were 21 participants across 5 focus groups. A total of 10 clinicians participated across 2 clinician-only focus groups, representing the following professions: geriatric medicine (n=2, 20%), neurology (n=3, 30%), social work (n=2, 20%), occupational therapy (n=1, 10%), psychology (n=1, 10%), and psychiatry (n=1, 10%). Clinician degrees included Occupational Therapist (1/10, 10%), Licensed Psychologist (1/10, 10%), Master of Social Work (1/10, 10%), Licensed Clinical Social Worker (1/10, 10%), Licensed Independent Clinical Social Worker (1/10, 10%), Doctor of Medicine (6/10, 60%), Doctor of Science (1/10, 10%), and Doctor of Philosophy (PhD; 3/10, 30%). Participants reported an average of 10.95 (SD 10.20; range 2-20) years of experience in dementia care. The majority were women (6/10, 60%) and White (9/10, 90%) individuals.

A total of 4 caregivers participated in a caregiver-only group. Participants were primarily women (4/4, 100%), and half identified as White (2/4, 50%) individuals.

A total of 3 person living with dementia–caregiver dyads (n=6) and 1 solo caregiver (ie, the corresponding person living with dementia was unable to attend) participated across 2 dyad focus groups. Persons living with dementia were primarily men (2/3, 67%) and White (2/3, 67%) individuals. Caregivers were primarily women (4/4, 100%), and half identified as White (2/2, 50%) individuals.

#### Procedure

The sequence of focus groups was clinicians (n=2), person living with dementia–caregiver dyads (n=2), and caregiver-only (n=1) group. A PhD-level clinical psychologist with experience in dementia care and qualitative research led 4 focus groups with clinicians and person living with dementia–caregiver dyads. During dyadic focus groups, the facilitator made every effort to ensure active participation from persons living with dementia, including seeking collateral information from dyad members (eg, confirming caregiver statements with persons living with dementia), asking binary questions (eg, yes or no), rephrasing questions when needed, and making behavioral observations with verbal confirmations (eg, “I noticed you perked up when I mentioned music, do you enjoy listening to music?”). Two trained bachelor-level research assistants coled the caregiver-only group. None of the interviewers had previous relationships with the participants. Group sessions lasted an average of 54 (range 51–57) minutes. All focus groups were completed virtually over secure Zoom, audio recorded, and transcribed.

During each focus group, participants responded to semistructured interview questions targeting activity preferences, technology preferences, usability considerations, and safety concerns; refer to Multimedia Appendix 1 for example focus group questions. In addition, participants provided feedback on a series of mock-up videos (ie, disjointed visuals designed to elicit conceptual feedback and ideas for intervention modules), including passive nature views (ie, waves crashing and Northern lights), guided relaxation with nature background (ie, night sky with voice-over), and abstract color flow (ie, colorful viscous swirl). Mock-up videos were previously used by the design team to inform the development of other IVET platforms targeting nondementia populations. Videos were chosen to provide insight into design features that aligned with our proposed mechanistic targets of sustained attention, positive emotions, and active engagement [42]. Participant feedback was designed to elicit preferences, usability considerations, and safety concerns (eg, “What problems do you see with this experience?” and “Do you foresee any concerns for safety or otherwise?”). Consistent with an iterative process of design, videos and interview questions were updated between each round of focus groups, such that videos or topics that elicited concerns for usability (eg, querying about the utility of sensory kits of household items, such as spices, to accompany videos, which elicited concern for feasibility) or safety (eg, abstract color flows, which elicited concern about increasing agitation or confusion) were omitted, and others were added (eg, new nature scenes with multiple points of view).

During each focus group, 1 or 2 bachelor-level research assistants observed the interviews and completed live field notes using a standardized rapid data analysis (RDA) template created for use in this study; refer to Multimedia Appendix 1 for the RDA template. At the conclusion of each focus group, all facilitators and observers completed reflective field notes using a standardized form with categories similar to those in the RDA template.

### Phase 2: Beta Testing Workshops

#### Participants

A total of 9 person living with dementia–caregiver dyad (n=18) participants were recruited across 8 beta testing workshops. Dyads were recruited separately for phases 1 and 2; none of the dyads who participated in phase 1 participated in phase 2. However, dyads enrolled in phase 2 were allowed to participate in multiple workshops if desired. Of the 9 dyads, most (6, 67%) participated in 1 workshop and 3 (33%) participated in 2 workshops; however, none participated in >2 workshops. Participants ranged from 1 to 4 dyads per workshop, with the majority consisting of 1 dyad (6/8, 75% workshops).

Across workshops, 100% (18/18) of participants identified as White individuals. Most persons living with dementia (7/9, 78%) identified as male, and most caregivers (7/9, 78%) identified as female. Caregivers provided proxy reports of dementia diagnoses, per their understanding of the medical records of the persons living with dementia, which included Alzheimer disease (2/9, 22%), unspecified dementia (4/9, 44%), Lewy body dementia (1/9, 11%), primary progressive aphasia (1/9, 11%), and frontotemporal dementia (1/9, 11%). Given that this study did not access medical records, caregiver reports were not confirmed, and the stage of illness was not reported. Multiple dyad relationship types were noted, including spousal (4/9, 44%), sibling (1/9, 11%), and adult child (1/9, 11%) caregivers.

#### Procedure

Beta testing workshops were led by members of the industry partner design team, with 1 to 2 trained research assistants in attendance to assist dyads in navigating the platform and to complete live field notes for RDA. All workshops were completed virtually over secure Zoom and recorded for the research team to review after session completion (eg, detailed feedback and behavioral observations). If multiple dyads engaged in 1 session, breakout rooms were used to enable private viewing experiences. Beta testing workshops lasted an average of 63 (range 34-103) minutes.

Workshops were completed via Zoom. At the start of each workshop, the design team shared a website link to the initial IVET intervention prototype with participants. Dyad participants were instructed to share their screen after opening the website. A workshop facilitator then prompted dyads to navigate the intervention both freely (eg, “As you explore the area, please share your thoughts out loud”) and with specific instructions (eg, “Can you figure out how to navigate back to the home screen?”). Participants started by freely navigating the prototype, providing live feedback on usability and acceptability (eg, confusing instructions and enjoyable features). Then, participants were presented with 1 to 3 modules to navigate, guided by facilitator prompts that primarily focused on usability and acceptability (eg, “Are there specific features on the island that drew your interest?” “What are your initial thoughts on the visuals of the activity?” and “Was navigating around the island intuitive or challenging?”). Dyads could choose the module they were most interested in navigating. In some situations, the second or third module was chosen for the dyad to ensure that feedback was gathered for each module. At the conclusion of each workshop, caregivers and persons living with dementia independently responded to quantitative measures assessing the perceived feasibility and usability of the platform. As workshops progressed, feedback became more targeted (eg, preference for specific images) than in earlier workshops (eg, preferred level of feedback from the platform), moving toward an initial prototype.

During workshops, research assistants completed observational field notes using a standardized template created for use in this study; refer to Multimedia Appendix 1 for the template. Field notes were used as part of RDA to inform intervention refinements between each workshop.

### Measures

Usability was assessed using the System Usability Scale (SUS) [46]. The SUS consists of 10 items evaluating the perceived usability of technological platforms, including websites. The SUS is the most widely used scale to assess usability for technology-based interventions in the context of dementia, but self-report challenges may exist with more advanced cognitive limitations [47]. However, the SUS is primarily applied to persons living with dementia with mild cognitive impairments and lacks psychometric data across the full spectrum of dementia (ie, limited data available in advanced stages of dementia) [47]. Furthermore, the SUS uses 5-point Likert scale for response options [48,49]. Therefore, following best practices to reduce cognitive load and ensure that persons living with dementia could meaningfully participate, we adapted this scale to reflect yes or no response categories [47,50]. This decision increased the likelihood that persons living with dementia in this study could provide feedback independently.

A total of 3 additional study-specific feasibility questions targeted entertainment, safety, and control. These items were adapted from previous technology-based intervention studies with participants with mild cognitive impairment and their caregivers [48] and provided feedback salient to our conceptual model (eg, engagement and positive affect) and design aims (eg, safety). These items were also asked in a yes or no format to increase inclusivity for persons living with dementia.

### Intervention Prototype

**Figure 2:**
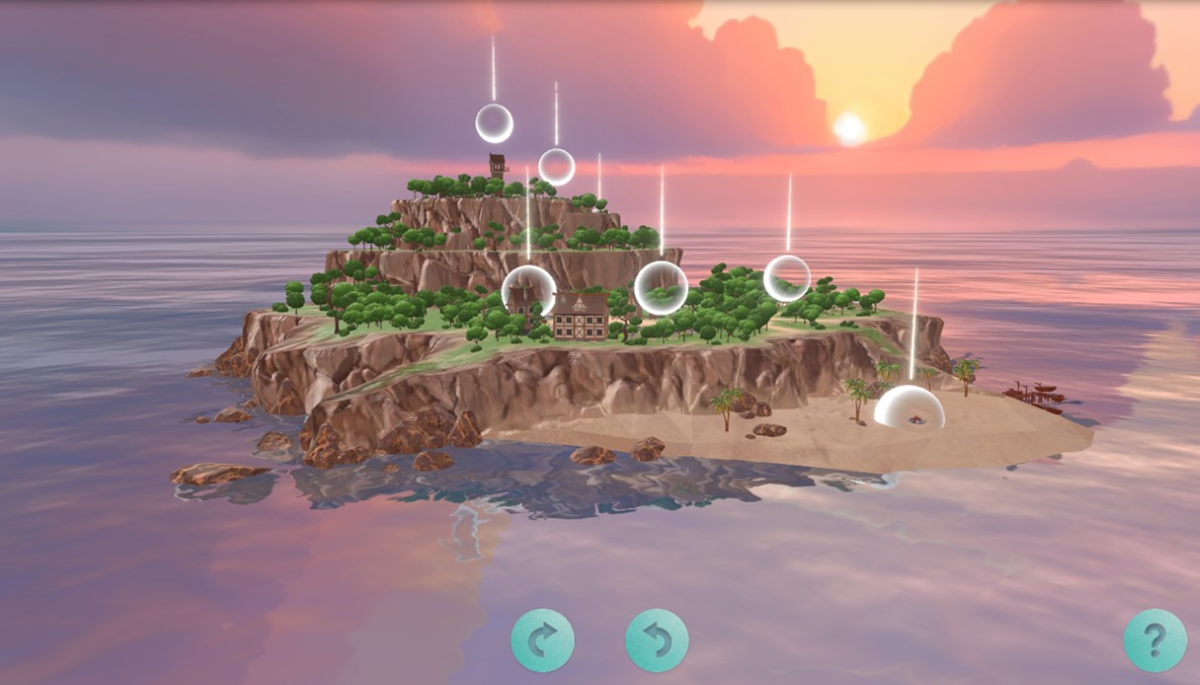
The Toolkit for Experiential Well-Being in Dementia (Isle of TEND) home screen.

Activities outlined in Phase 1: Focus Groups and Phase 2: Beta Testing Workshops sections culminated in the development of an initial prototype of a web-based dyadic IVET intervention, referred to as the Toolkit for Experiential Well-Being in Dementia (Isle of TEND). The Isle of TEND website contained a home page (Figure 2) with the ability to navigate to 8 modules, which contained different shared activities; refer to Multimedia Appendix 2 for an overview of modules. Consistent with our conceptual model [42], each module in the Isle of TEND featured a shared activity intended to promote sustained attention, positive emotions, and active engagement with the intervention. The intervention was designed to be used by both persons living with dementia and caregivers together in the home.

### Data Analysis

This study used RDA as an analytic framework to identify themes from focus groups and workshops. RDA is used to quickly identify themes in qualitative data [51]. Rather than written transcriptions, this technique relies on live coding directly from interviews, such as taking observational notes during interviews or while listening to an audio recording [52-54]. For this study, RDA was chosen to identify specific feedback from participants related to preferences, usability, and safety that could guide prototype development and refinement. By quickly capturing this information, our research team was able to communicate findings back to our design team to iteratively respond to feedback and update the intervention prototype to gather more feedback from users.

Consistent with previous studies using RDA [55], we compiled RDA template notes from 2 to 3 team members (ie, 1 PhD-level clinical psychologist and 2 bachelor-level research assistants) into a single matrix for each focus group. Data from this matrix were used to guide discussions with the design team and informed design decisions toward initial prototype development.

At the conclusion of the study, matrices were combined and used to facilitate thematic analysis of focus groups and workshops separately. Focus group data were analyzed as a single dataset (ie, clinician, caregiver, and dyad focus groups analyzed together), such that we identified themes that emerged across groups. We did not compare themes across populations, as this was not consistent with the aims of this study. Two research assistants (ER and AT) trained in qualitative RDA methods independently reviewed matrices to identify codes. Coders met on multiple occasions during the coding process to resolve discrepancies. Codes were grouped by content to form themes. The thematic structure was iteratively developed and confirmed with a meeting between coders and a clinical psychologist on the study team with experience in dementia care (EP). After a thematic structure was agreed upon, the research assistants (ER and AT) reviewed transcripts to identify salient quotes for each thematic category. Both focus groups and workshops followed the same analytic steps. However, transcripts were only reviewed for focus groups because the data collected during workshops produced fewer exemplar quotes (eg, workshops contained more “thinking aloud” methods or behavioral observation, making data less relevant for exemplar quotations).

## Results

### Phase 1: Focus Groups

Three themes emerged from focus groups with clinicians, caregivers, and dyads: (1) designing flexibly to allow users to tailor the intervention experience to their own environmental context and circumstance, (2) designing with the dyad’s clinical and relational needs in mind, and (3) accounting for illness and aging-related challenges in the design.

#### Theme 1: Designing Flexibly to Allow Users to Tailor the Intervention Experience to Their Own Environmental Context and Circumstance

The ability for users to individualize their experience emerged as an important design feature to increase acceptability and usability as well as to empower choices among users. Participants noted that the option to individualize or tailor intervention content could help accommodate a larger range of users based on their access to and comfort navigating technology. In addition, participants recommended that the IVET intervention be accessible via various platforms. For example, a clinician noted the following:

[Consider] an app that is free, that can download the materials into [users] cell phone or their device so it doesn't depend on the Internet connection...and maybe having the option of audio only or audio plus visual.

Participants also noted the importance of developing stimuli that were familiar to and pleasant for participants of various backgrounds, cohorts, and identities to promote individualization. Furthermore, participants noted that familiar stimuli may especially increase comfort, enjoyment, engagement, and safety for persons living with dementia. A caregiver highlighted the potential benefits of familiar stimuli:

Having an inspirational waterfall isn’t necessarily going to put me at ease, but maybe hearing my family’s familiar voice, or the clattering of my own kitchen, or having something that individualizes this to me would.

Similarly, a clinician highlighted the need for inclusivity as follows:

I just wonder if the salience of some of the images might be different depending on the cultural groups. The aurora borealis is beautiful, but for maybe people who [are] not familiar with that part of the of the world...what are the scenes, the sounds that are most salient and comforting to that individual.

Of note, related to the environmental context of an at-home IVET intervention, caregivers discouraged the use of a proposed sensory kit of household materials, citing feasibility issues with the ability for a range of users to locate these materials in their home (eg, spices).

#### Theme 2: Designing With the Dyad’s Clinical and Relational Needs in Mind

Participants noted that designing an intervention with a specific purpose and orienting users to a rationale for using the intervention may increase usability. Specifically, participants suggested recommendations for appropriate times and intended outcomes of use (eg, using when calm and avoiding when agitated). For example, a clinician noted the potential clinical utility of an IVET intervention:

I think that [the calming sounds are] good, and it appeals to an inherent positive emotional experience which could also aid caregivers when they’re trying to prep for redirection or transition. Basically, if we're trying to get somebody to do something that they may not necessarily want to do we’re far more likely to be able to if a person is in a positive emotional state versus a negative one and so these types of things could aid in that process of prepping a patient for a transition.

Caregivers believed that they would likely need to be present to initiate the intervention as well as to guide the persons living with dementia through some of the modules. For example, a caregiver noted the following:

[Caregivers] would have to be there to sort of guide [Person living with dementia] or kinda help them figure out what [the intervention] is.

Participants were skeptical that an IVET intervention could be engaging for both dyad members, noting challenges of achieving sustained attention and immersion for both persons living with dementia and caregivers. This is highlighted by a caregiver who stated the following:

[Person living with dementia] loses interest pretty quick, and I think sometimes he has trouble following the storyline. So, it would be hard to find something that would be suitable for both of us, cause we’re kind of in very different places now. And that’s one of the things that’s difficult is we don’t have as many shared experiences as we used to.

Participants recommended structured stimuli to quickly capture and sustain the attention of users, which they noted could be challenging in the context of dementia. For example, a caregiver stated the following:

Without having a clear beginning or end, [the person living with dementia] might get bored or discouraged, it needs to have more structure.

Furthermore, participants emphasized that the IVET intervention would need to be designed to sustain attention and immersion without overstimulating or confusing users with cognitive challenges.

#### Theme 3: Accounting for Illness and Aging-Related Challenges in the Design

Participants noted the importance of ensuring the reduction, rather than an unintentional exacerbation, of behavioral and psychiatric symptoms of dementia, particularly agitation and hallucinations. This concern is highlighted by a clinician, who stated the following:

Dementias with any psychiatric component or psychotic features, paranoia, delusional thinking...you always want to be careful about providing stimulation when we don’t know where that may lead the patient’s train of thought.

**Figure 3:**
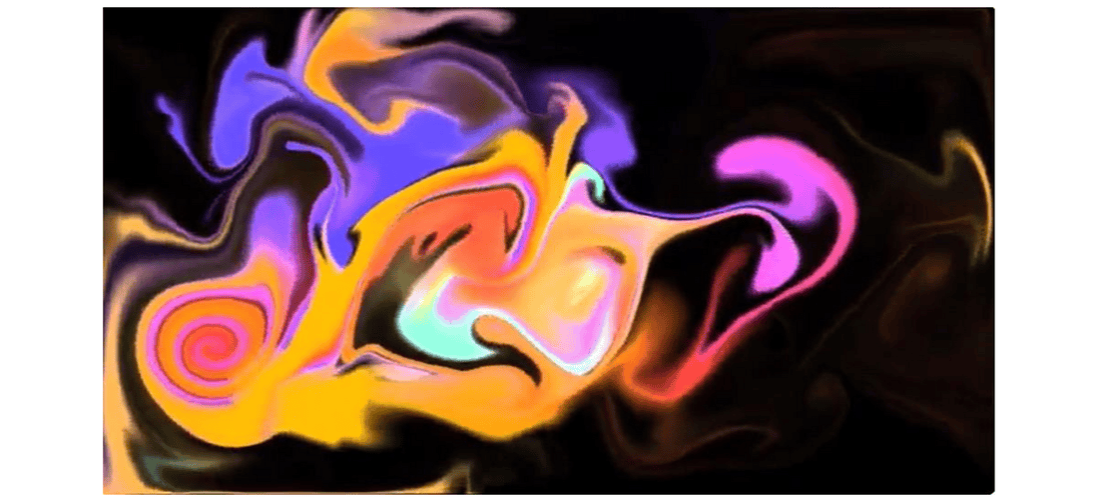
Swirling color scheme video.

There were specific stimuli that participants recommended avoiding, such as 1 video with a moving (ie, swirling) bright color scheme against a black background (Figure 3), which participants suggested could potentially exacerbate psychiatric symptoms. For example, a clinician stated the following:

I think patients would not like [the swirling color video], they have a lot of visual spatial misperceptions.

Furthermore, participants cautioned against dark backgrounds, which might increase psychiatric symptoms and confusion or disorientation. For example, a caregiver noted the following:

My husband is really paranoid, he thinks people are out to get him. I was thinking of the lighting, if there is darkness that might increase his paranoia.

Another caregiver noted the following:

The edges of my video were very dark. So, it gives you almost like that movie theater feel to it, which I worry, if you're immersed in something too much, do you have less grasp on here and now where you are.

In addition, as noted across multiple themes, participants relayed that certain cognitive symptoms, especially attentional deficits and apathy, could pose a challenge to engagement and usability. One clinician stated the following:

I wonder if in some instances this [mock-up videos] may be a little too unstimulating to capture the patient’s attention, unless they are really already very calm and wanting to be calm.

Another clinician stated the following:

for images...it might be really, really simple ones, the less complicated you make it, the better received because [person living with dementia] cognitive abilities and processual abilities are impaired.

Participants also noted that visual clarity was an important design feature and recommended that the IVET intervention accommodate for age-related changes and pathologies more common with older age. For example, 1 caregiver stated the following:

There’s something about lighting that I would pay attention to for elders...I have another elder in my life who has glaucoma and she can't see things in depth of perception and other things.

### Phase 2: Beta Testing Workshops

#### Qualitative Results

Two main themes emerged from beta testing workshops with dyads that informed intervention refinement: (1) more feedback to guide using the intervention and (2) more variety of visual and auditory experiences.

#### Theme 1: Increasing User Support Through More Feedback

Participants noted that more purposeful or goal-directed actions within intervention modules could reduce confusion during use. When engaging with the intervention, caregivers and persons living with dementia frequently inquired about the end goal of modules, especially when feeling “lost” or “stuck.” Relatedly, participants recommended more instructions, which, per observation, appeared to be related to participant concerns about making an error or doing the experience “wrong.” Together, an open-ended module without clear directions, which was initially developed to increase exploration and reduce the need for caregiver instructions, actually increased confusion and perceived misuse of the intervention by users.

Furthermore, participants voiced the need for continuous instructions throughout use to understand next steps, which was particularly relevant in modules that included multistep tasks. For example, a module that developed a poem based on typed responses to multiple prompts caused some confusion among users because dyads were unsure of the task they were being prompted to complete (eg, not knowing that prompts would generate a poem) or the end goal of the multistep process (eg, the need to press next and respond to another prompt to develop the full poem). In addition to more prompts, participants suggested having multiple methods of engagement, such as a speech-to-text function, to promote accessibility and usability.

#### Theme 2: Increasing the Variety of Visual and Auditory Feedback

Participants recommended increasing the presence and variability of responsive sounds, background music, and interactive visuals. Caregivers and persons living with dementia both noted that alternating music or background colors might reduce redundancy after multiple uses and make the intervention more engaging. In addition to increasing engagement, dyads noted that sound effects, especially calming nature sounds, improved their experience by creating a sense of calm while using the intervention.

### Quantitative Analysis

In general, users rated the intervention positively (Table 1). On the study-specific feasibility scale, 100% (11/11) of caregivers and 67% (6/9) of persons living with dementia (n=2, 22% were unsure or did not respond) felt that the program was safe to use, 82% (9/11) of caregivers and 100% (9/9) of persons living with dementia felt that the program was entertaining, and 82% (9/11) of caregivers and 89% (8/9) of persons living with dementia felt that the program helped them “feel in charge of their own experience.” On the SUS, 82% (9/11) of caregivers and 89% (8/9) of persons living with dementia thought that the program was “easy to use,” and 73% (8/11) of caregivers and 78% (7/9) of persons living with dementia “felt very confident using the program” (n=1, 11% were unsure or did not respond).

**Table 1:** Feasibility and usability results of beta testing workshops.

	Caregivers, (n=11), n (%)				Persons living with dementia (n=9), n (%)		
	Yes	No	Unsure or unable to answer	Yes		No	Unsure or unable to answer
“I think that I would like to use this program.”	7 (64)	2 (18)	2 (18)	6 (67)		2 (22)	1 (11)
“I found the program unnecessarily complex.”	1 (9)	10 (91)	0 (0)	1 (11)		8 (89)	0 (0)
“I thought the program was easy to use.”	9 (82)	2 (18)	0 (0)	8 (89)		1 (11)	0 (0)
“I think that I would need the support of a technical person to be able to use this program.”	4 (36)	7 (64)	0 (0)	3 (33)		4 (44)	2 (22)
“I found the various functions in this program were well integrated.”	9 (82)	2 (18)	0 (0)	6 (67)		3 (33)	0 (0)
“I thought there was too much inconsistency in this program.”	2 (18)	9 (82)	0 (0)	2 (22)		5 (56)	2 (22)
“I would imagine that most people would learn to use this program very quickly.”	7 (64)	3 (27)	1 (9)	4 (44)		0 (0)	5 (56)
“I found the program very cumbersome to use.”	0 (0)	11 (100)	0 (0)	1 (11)		5 (56)	3 (33)
“I felt very confident using the program.”	8 (73)	2 (18)	1 (9)	7 (78)		1 (11)	1 (11)
“I needed to learn a lot of things before I could get going with this program.”	1 (9)	10 (91)	0 (0)	2 (22)		4 (44)	3 (33)
“Was this program able to entertain your relative and you?”	9 (82)	2 (18)	0 (0)	9 (100)		0 (0)	0 (0)
“Did you feel that the program was safe to use for your relative and you?”	11 (100)	0 (0)	0 (0)	6 (67)		1 (11)	2 (22)
“The program helped my relative feel in charge of their own experience.”	9 (82)	2 (18)	0 (0)	8 (89)		1 (11)	0 (0)

### Feedback-Design Integration

Throughout intervention development, our academic-industry team met weekly to discuss results from focus groups and workshops and translate findings into pragmatic design elements. Often, the research team helped provide the context of findings within the illness context of dementia, as well as guide a systematic approach to intervention design that would prepare the team for a future, scientifically rigorous clinical trial. The industry design team often discussed the possibilities and limitations of an IVET intervention delivered with minimal equipment (ie, via a computer or tablet). These perspectives led to productive discussions about incorporating user feedback into intervention design. Together, this iterative process led to the end product of an IVET intervention ready for initial feasibility testing (NIH stage 1B) that was deemed safe and usable by end users (ie, person living with dementia–caregiver dyads). Feedback and design decisions are provided in Table 2.

**Table 2:** User feedback and design team responses.

User feedback			Design feature
**Focus groups: initial prototyping**			
	Implement choice-based activities when possible.	The team decided on a web-based IVET^a^ format so that users could guide themselves through the platform. The intervention was user driven, and users chose from various modules in the intervention, deciding the type and length of activity within each module.	
	Establish the ability to adjust audio or visuals depending on preferences.	Developers enabled access to the intervention on the user’s own tablet or computer so that users could adjust the volume and brightness to their preference.	
	Include visuals or audio that are appealing and familiar to diverse populations.	The design team developed 8 different modules, including modules set in a forest, an ocean, a beach, and a lighthouse.	
	Integrate instructions for the intervention.	The design team included text instructions on the home screen to orient users when they logged into the intervention. The instructions reviewed how to navigate through the home screen and the modules.	
	Intervention can be used among people with and without cognitive impairment.	Modules were “error-free” so that users were unable to “fail” or choose the “wrong” option, halting the experience; regardless of user choice, the experience continued.	
	Include stimuli that are reactive to touch or hand or arm movement.	Developers created two activities, which accessed the device’s camera to track and react to hand movement: (1) Sound Garden, which used hand movement to control the music pitch and volume in a garden scene, and (2) Art Studio, which used hand movement to create brush strokes on the screen to reveal an art piece.	
	Design modules that are grounded in reality (familiar structure and clear beginning and ending).	The team set the intervention in recognizable locations, such as a waterfall, a lighthouse, and a beach. The game had a clear beginning with text instructions when users logged on.	
	Avoid abstract images and darkness in visuals.	The team removed abstract “swirling colors” stimuli. The team discarded a video set in a dark forest environment and brightened the colors in the remaining modules.	
**Beta testing workshop: prototype refinements**			
	Clarify the end goal or purpose of the intervention. Include more goal-directed aspects to the intervention.	The design team added an introduction slide clarifying that the intervention was designed to create “shared adventures” between the dyad and “engage the senses and spark imagination.” The team included a “help” button across all modules to guide users.	
	Implement different methods of engagement, such as a speech-to-text option instead of typing.	Developers included a speech-to-text function in modules that required users to type. These activities accessed the device microphone to capture spoken responses from the dyad and convert them into text. To increase engagement, developers created custom soundscapes for scenes and changed them based on the visual stimuli presented.	
	Add more instructions and audio-to-text instructions.	Developers added a module description when users hovered their mouse over each module location. The team added step-by-step tutorials, which isolated different interactive aspects of the experience when users clicked on each module. In addition, the team added voice-over to text as an accessibility option to all introductions and guides.	
	Increase the visual stimuli throughout use to reduce redundancy.	The team increased the visual options in modules throughout the experience by creating more artwork and audio clips. For example, in the Storytelling module Campfire Cove, the team increased the number of available pictures to choose from and created an accompanying story.	
	Include visual and audio stimuli, such as animation or music, in modules that lacked sounds or featured static images. Add calming music or nature audio when possible.	The design team added nature-based animation and sound on the home screen, including auditory and visual waves, so that when logging on, users were greeted with these stimuli. In addition, the design team added nature-based sounds to the background of module instructions, such as birds chirping and “calming” music.	

^a^IVET: immersive virtual environment technology.

## Discussion

### Principal Findings

Following a multiphasic user-centered design framework with dementia-specific considerations [38], this study used focus groups and user workshops to develop and refine the initial prototype of a dyadic IVET intervention to support the emotional health of person living with dementia–caregiver dyads (ie, the Isle of TEND). The intervention was rooted in our conceptual model of shared activities that can promote sustained attention, positive emotions, and active engagement among both dyad members simultaneously [42]. The Isle of TEND is now ready for feasibility testing, consistent with NIH stage 1B.

### Design Considerations

#### Overview

Across focus groups and workshops, we identified several key considerations for designing an IVET intervention for person living with dementia–caregiver dyads. Importantly, we did not constrict our intervention to a specific stage of illness. Rather, we engaged users, including those with cognitive limitations as evidenced by UBACC scores, and other knowledge holders who could provide feedback relevant to users with varying degrees of cognitive ability. This approach allowed us to capture multiple voices and perspectives to ensure that we are designing for all persons living with dementia, including those often not considered during the design stage due to significant cognitive impairment.

#### Personalization and Variety of Experiences

Consistent with previous research on technology-based interventions with persons living with dementia [56,57] and activity engagement in them [58], personalization and variety of experiences emerged as key design features for our intervention. This feedback is not surprising, given the heterogeneity of identities, preferences, and lived experiences of dyads managing dementia. Increasing the number of novel or rotating visuals and sounds in each module allowed for personalization and helped to reduce boredom with repeated use. Our design team was cautious to only make slight variations in activities (eg, sounds or storytelling prompts) to reduce boredom without increasing confusion. Consistently, participants noted that nature-based sounds and animation, such as waves or chirping birds, evoked positive emotions. Therefore, our design team focused on nature scenes for many of our modules. Previous research with persons living with dementia supports that nature sounds introduced through technology, referred to as a soundscape, are perceived positively with minimal safety concerns [59,60].

#### Optimizing Autonomy and Choice While Sustaining Ease of Use

Feedback related to personalization highlighted an important component of designing in dementia: optimizing autonomy and choice while sustaining ease of use. In addition to increasing acceptability, the ability to tailor interventions to personal preferences may create an opportunity for persons living with dementia to have more control over their user experience. This was important for both members of the dyad to promote a sense of accomplishment, autonomy, and confidence and, ideally, allow the dyad to break the care recipient-caregiver script, enabling both members to feel as though they have ownership of their own experiences with the intervention. Previous technology-based interventions developed for persons living with dementia with mild cognitive impairments also note the importance of prioritizing user control [48]. Furthermore, in the context of dyads, increasing autonomy and choice for persons living with dementia means reducing the need for the caregiver to “deliver” the intervention. While refinements to our prototype are still needed to fully achieve this outcome, it is an important endeavor for dyadic interventionists in the context of dementia, as allowing for a break in the care recipient-caregiver script may produce positive emotional benefits for both dyad members across all stages of illness.

Users consistently provided feedback that some user-driven choices in our platform were too open ended (eg, coming up with a poem without prompts), which could lead to confusion and produce the opposite emotional experience for dyad members than intended (ie, frustration with being “wrong” and the perceived need for caregiver-directed behaviors). To address this balance, our design team attempted to provide ample instructions and feedback for use while not imposing “rules” (ie, “error-free” design) and promoting exploration. For example, several of our modules initially present users with directions for navigating the activity and then provide participants with open-ended (ie, pictures of different scenes) and forced-choice (ie, multiple choices for storytelling) decisions to guide their experience based on their needs and preferences. In addition to providing directions throughout the module, as recommended by users, we developed responsive features to reduce cognitive load and avoid frustration. For example, following effective communication strategies in the context of dementia [61], if a dyad did not respond to a multiple-choice prompt within a period of time, a forced-choice option appeared (ie, this or that). Most users with dementia rated that they felt in control of their own experience and confident in using the Isle of TEND, which may be a product of these design features.

#### Maintaining Engagement Among Cognitively Mismatched Users

We set out to design an IVET intervention that can maintain sustained attention and engagement among cognitively mismatched dyad users while at the same time not overwhelming the persons living with dementia or underwhelming the caregivers. Participants recommended that to achieve this goal, we needed to have a clear structure, continuous feedback, and novel stimuli, which are consistent with theory (eg, flow) [62]. In response to these recommendations, our design team developed a home screen with an introduction to the IVET intervention, tutorials and instructions for each module, and a navigation guide that appears constantly. Furthermore, our design team developed several modules that are reactive to touch or movement. For example, 1 module, the Art Garden, accesses the device camera and uses hand movement as “brushstrokes” to paint a picture. Despite these features designed to promote sustained attention and engagement, user feedback in the workshops was still mixed regarding the ability to capture and sustain engagement across cognitively mismatched dyads and reduce caregiver-directed behaviors (eg, caregiver reiterating directions). However, it is important to note that 82% (9/11) of caregivers and 100% (9/9) of persons living with dementia still found the intervention to be entertaining. The field still needs to address the important topic of designing for mismatched cognitive abilities, as there are potential clinical benefits associated with shared engagement for person living with dementia–caregiver dyads managing dementia throughout the course of illness (eg, emotional closeness and relaxation). It is possible that design teams need to attend to more nuanced interactions between users and platforms to evaluate common elements of sustained attention and engagement across users with varying cognitive abilities (eg, movement).

#### Not Exacerbating Behavioral and Psychiatric Symptoms of Dementia

Regarding safety concerns, participants flagged specific stimuli that could potentially lead to distress or exacerbate behavioral or psychiatric symptoms that accompany certain types of dementia. In particular, participants recommended avoiding abstract stimuli, bright colors, or moving objects against a dark background, as these could potentially increase adverse events (eg, agitation) in more advanced stages of dementia. These images may relate to overstimulation or confusion, which increases behavioral and psychiatric disturbances in the context of advanced dementia when persons living with dementia have increased vulnerability to environmental stressors [63]. Users suggested that, in general, simpler and easily recognizable images may be better received by both persons living with dementia and caregivers. Participants also identified dark or shaded visuals as potentially problematic (eg, blurriness and glare) for users with age-related or other visual impairments. In response to the feedback, the design team removed visuals and modules that relied on limited lighting and highly abstract images. Of note, there were no observed exacerbations of dementia-related behavioral or psychiatric symptoms during workshops.

### Limitations

This initial intervention development study has several limitations to consider. First, our convenience sample of caregivers, dyads, and clinicians consisted of a majority of White participants. In future pilot testing, we will ensure that participants are more representative of the US population. Second, we did not administer cognitive assessments to describe the level of cognitive impairment of our sample. Therefore, we cannot empirically comment on the cognitive profile of participants with objective measurement, including the severity of cognitive impairment. This study used self-reports from persons living with dementia and caregivers to capture the presence of dementia, and the UBACC provides additional insight into the presence of cognitive limitations inhibiting the ability to provide autonomous consent. Indeed, dementia research must balance participant burden, the reality of difficulties obtaining a dementia diagnosis, and study goals. In line with our broader goal of offering TEND as a free intervention for individuals in community settings, we included participants with varying levels of cognitive ability, as reflected by the fact that only 17% (2/12) of our sample was able to provide independent consent. To circumvent challenges with reporting on quantitative surveys for those with more advanced dementia, our team took multiple steps to support persons living with dementia in reporting their own outcomes, including using binary scales and reading questions aloud. However, these methods may have reduced the validity of our measures, and we still cannot be sure that all items were fully comprehended. Finally, we chose RDA over other forms of qualitative analysis. For this study, prioritizing a quick summary of information and relaying this to our design team consistent with RDA was necessary, but this analytic approach may also be more susceptible to researcher bias.

### Conclusions

The result of this multiphasic user-centered design study with an academic-industry design team was the development of an initial dyadic IVET intervention for persons living with dementia and their caregivers to use together at home. On the basis of our conceptual model, we aimed to promote sustained attention, positive emotions, and active engagement in both dyad members to increase relationship satisfaction and reduce psychosocial distress. In this study, we highlight early intervention development with a focus on designing for usability and safety. While some of our design considerations may be helpful for future investigators and design teams focusing on dyads managing dementia, we also recognize that there are gaps that still need to be filled, such as promoting mutual engagement among cognitively mismatched dyads.

## Appendix

Multimedia Appendix 1 Example questions from phases 1 and 2 and rapid data analysis template.

Multimedia Appendix 2 Toolkit for Experiential Well-Being in Dementia (Isle of TEND) modules.

## Abbreviations

Isle of TEND: Toolkit for Experiential Well-Being in Dementia
IVET: immersive virtual environment technology
NIH: National Institutes of Health
RDA: rapid data analysis
SUS: System Usability Scale
UBACC: University of California, San Diego Brief Assessment of Capacity to Consent Questionnaire
