# Supplementary material for: Developing a Dyadic Immersive Virtual Environment Technology Intervention for Persons Living With Dementia and Their Caregivers: Multiphasic User-Centered Design Study
Source: JMIR Aging. 2025 May 21;8:e66212. doi: 10.2196/66212 (PMC12138290; doi:10.2196/66212)
Supplement: Multimedia Appendix 1 [file aging_v8i1e66212_app1.docx]

**Example Focus Group Questions:**

1. Are there shared activities that you no longer do or wish you did more of together?
2. Could something like this grab your attention? If not, what could be changed to be more engaging?  [referencing mock-up videos]
3. How might you use an experience like this in your day-to-day life? Would you use something like this? [referencing mock-up videos]
4. What problems do you see with this experience? [referencing mock-up videos]

**Rapid Data Analysis Template - Clinician Focus Groups**

| **Reactions to Videos** | | | |
| --- | --- | --- | --- |
| General Reactions - Positive: | | General Reactions - Negative: | |
| Suggestions for Improvements: | | | |
| Video: Beach Scene | Video: Northern Lights | | Video: Waterfall |
| Video: River | Video: Island | | Video: Patterns |
| Other reactions: | | | |
| Reflections: | | | |

| **Relevance of Videos** | | | |
| --- | --- | --- | --- |
| Relevance to Clinicians: | Relevance to PLWD: | | Relevance to CGs: |
| Variability – Early stages of dementia: | | Variability – Later stages of dementia: | |
| Variability – Dementia subtype | | Variability – Dementia symptomology | |
| Other factors related to relevance: | | | |
| Reflections: | | | |

| **Adverse Events and Safety** | |
| --- | --- |
| General safety considerations for PLWD: | |
| Safety considerations by dementia subtype: | Safety considerations by dementia symptoms: |
| Safety considerations by context: | Safety considerations for CGs: |
| Video components that could lead to discomfort: | Indicators of discomfort in PLWD: |
| Other factors related to safety: | |
| Reflections: | |

| **Overall Reflections** |
| --- |
|  |

**Rapid Data Analysis Template – Caregiver and Dyad Focus Groups**

| **Shared Activities** |
| --- |
| Joy & Meaning: |
| Attention/Engagement: |
| Sense of Calm: |
| Other Activities: |

| **Video Feedback** |
| --- |
| First Impressions: |
| Positives: |
| Ability to Grab Attention: |
| Utility- Everyday Use: |
| Together vs. Separate Usability: |
| Feasibility of Sensory Items: |
| Negatives: |
| Specific Suggestions for Improvement: |
| Other Feedback: |

| **Overall Reflections** |
| --- |
|  |

**Rapid Data Analysis Template – Beta Testing Workshops**

| **Feedback on Operation** |
| --- |
| Navigation around the platform: |
| Ability to follow directions and prompts: |
| Ability to enter and exit activities: |

| **Feedback on Specific Activity (1)** |
| --- |
| Specific features of interest: |
| Overall impressions of activity: |
| Challenges: |
| Thoughts on the visuals: |
| Improvements: |

| **Feedback on Specific Activity (2)** |
| --- |
| Specific features of interest: |
| Overall impressions of activity: |
| Challenges: |
| Thoughts on the visuals: |
| Improvements: |

| **Feedback on Specific Activity (3)** |
| --- |
| Specific features of interest: |
| Overall impressions of activity: |
| Challenges: |
| Thoughts on the visuals: |
| Improvements: |

| **Overall Reflections** |
| --- |
|  |
