# Supplementary material for: Developing a Dyadic Immersive Virtual Environment Technology Intervention for Persons Living With Dementia and Their Caregivers: Multiphasic User-Centered Design Study
Source: JMIR Aging. 2025 May 21;8:e66212. doi: 10.2196/66212 (PMC12138290; doi:10.2196/66212)
Supplement: Multimedia Appendix 2 [file aging_v8i1e66212_app2.docx]

*Table 1*. Preliminary iteration of Isle of TEND modules with descriptions.

| Isle of TEND Module | Description |
| --- | --- |
| Lighthouse Point  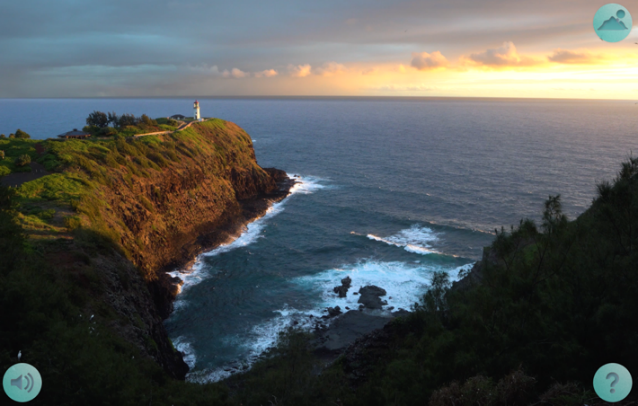 | Immersive sunrise experience on a cliffside overlooking a lighthouse and bird sanctuary, inducing awe and wonder. |
| Renewal Falls  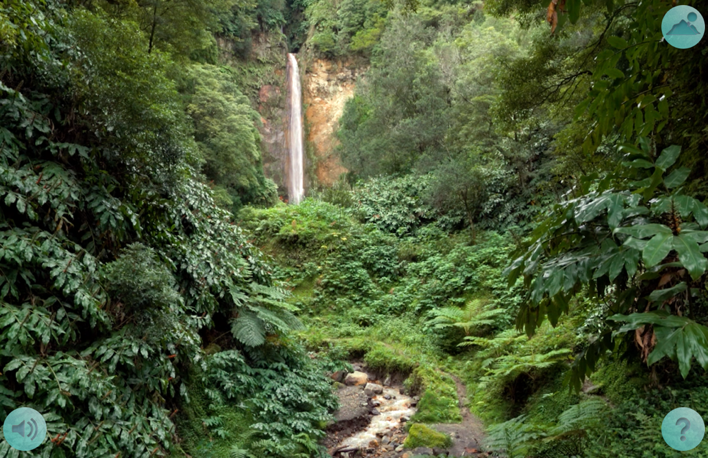 | Viewing point of a tropical rainforest with audio features, focus on rainforest sounds. Aimed to induce calm and gentle stimulation. |
| Observation Tower  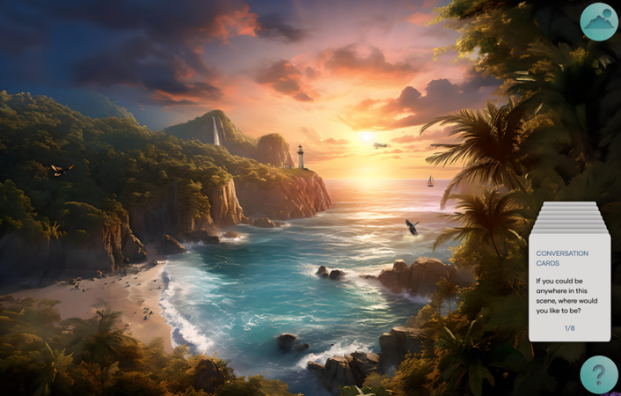 | Create a co-developed story through exploration – a binoculars icon allows for exploring new ‘chapters’ of each scene. Open-ended conversation cards to build off each other’s thoughts and ideas and develop a narrative. Each scene has a custom musical score that accompanies the visuals. |
| Art Studio  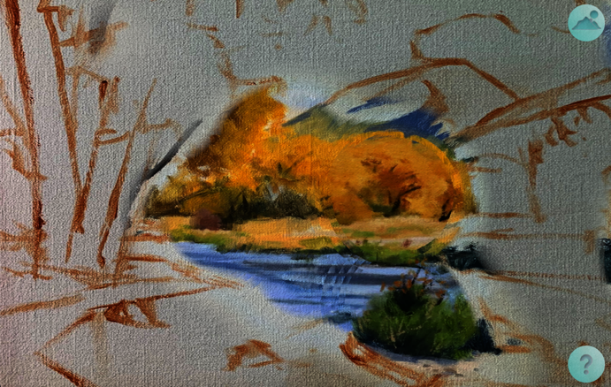 | Visual feature that turns body movement to art. Supports a mind-body connection. |
| Hillside Farms  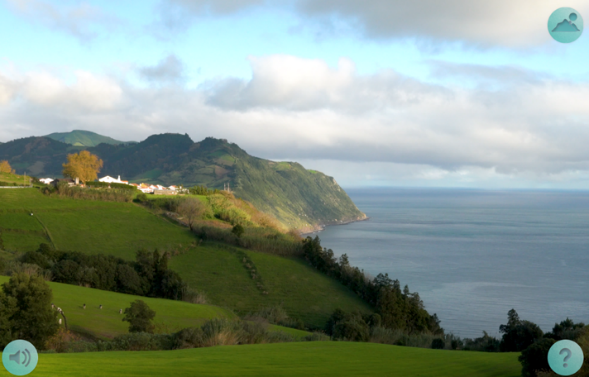 | Viewing point of rolling hills on the island and pastoral farm, aimed to induce calm and create a peaceful escape. |
| Sound Garden   | Visual and auditory feature that turns body movement into music. Supports a mind-body connection and promotes music making. |
| Poetry Palms  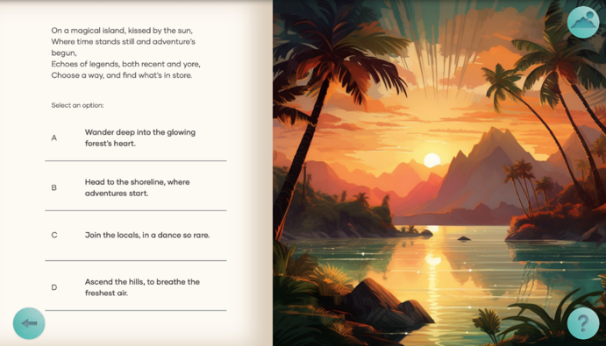 | Collectively create poems by selecting options that shape and change the next line of the poem, with changing art with each selection as well as a custom musical score. |
| Campfire Cove  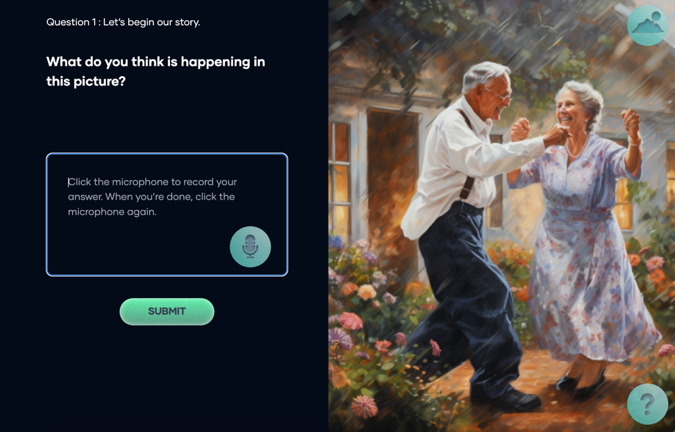 | Improvisational story building through prompts to mutually develop a story and narrative, set to a custom musical score. |
